# Epidemiologic Studies of Psychosocial Factors Associated With Quality of Life Among Patients With Chronic Diseases in Japan

**DOI:** 10.2188/jea.JE20110114

**Published:** 2012-01-05

**Authors:** Masayo Kojima

**Affiliations:** Department of Public Health, Nagoya City University Graduate School of Medical Sciences, Nagoya, Japan

**Keywords:** depression, alexithymia, risk factors, hemodialysis, rheumatoid arthritis

## Abstract

A link between affective disturbances and physical disorders has been suggested since the Greco–Roman era. However, evidence supporting an association between mind and body is limited and mostly comes from North America and Europe. Additional local epidemiologic studies are needed so that more evidence can be collected on effective treatments and health management. Epidemiologic studies of Japanese with rheumatoid arthritis (RA) and those on chronic hemodialysis examined the association between psychosocial factors and patient quality of life (QOL). Strong associations among depression, social support, and patient QOL were confirmed, which supports the findings of studies performed in Western countries. In addition, disparities between the perspectives of patients with RA and their doctors were observed. Alexithymia, a personality construct that reflects a deficit in the cognitive processing of emotion, had a stronger independent association with increased risk of 5-year mortality than did depression among patients with chronic hemodialysis. Physiological, biological, and psychosocial factors are associated and independently and interactively determine our health. Epidemiology is a powerful tool for identifying effective points of intervention, after considering all possible confounders. Future studies must clarify how health can be improved by using a psychosocial approach.

## NO HEALTH WITHOUT MENTAL HEALTH

The World Health Organization (WHO) defines health as “a complete state of physical, mental, and social well-being and not merely the absence of disease or infirmity”.^1^ Thus, health fundamentally consists of physical, psychological, and social factors. Links among affective disturbances, social factors, and physical disorders have been observed since the Greco–Roman era, and a 1990 editorial in *JAMA* maintained that the notion “that the brain can exert profound effects on the body” was “by no means a new idea”.^2^ Engel, a Nobel Prize-winning internist and psychiatrist, claimed that the development of chemistry and the physical sciences created a dominant biomedical model of disease that separated the mental and somatic aspects of disease, leaving no room within its framework for the social, psychological, and behavioral dimensions of illness.^3^ He proposed a biopsychosocial model to provide a design for action in “the real world” of health care. Recent advances in neurosciences, including brain imaging, have revealed a close link between psychological perception and physical responses.^4^ Moreover, the shift in the primary cause of death from infectious diseases to noncommunicable chronic diseases, such as heart disease, diabetes, and cancers, has strengthened the importance of a psychosocial approach to health management. The Global Health Risk Report by the WHO concluded that the most important global risks for mortality in the world are high blood pressure, tobacco use, high blood glucose, physical inactivity, and overweight and obesity.^5^ The biological approach has a limited capacity to reduce these health risks. Attending to the mind and individual social background is essential in the treatment of noncommunicable chronic diseases.^4^

The WHO now maintains that there is “no health without mental health”.^6^ The contribution of mental health disorders to disease burden has been increasing worldwide.^6^ According to the 2005 report of the WHO, 31.7% of all years lived with disability were attributed to neuropsychiatric conditions, among which depression was the leading cause.^7^ However, the association between mental disorders and disability remains underestimated.^6^ Affective disturbances can undermine long-term outcomes of physical disorders via behavioral and cognitive processes with specific and nonspecific biological responses.^8^ Conversely, physical disorders increase the developmental and prognostic risk of mental disorders. Thus, comorbidity complicates health problems and increases the difficulties of individual patients.

Although an association between mental and physical health disorders has been strongly suggested, most of the available evidence for this association has come from North America and Europe, and investigations assessing the prognostic effects of mental illness on health outcomes are rare.^6^ Psychosocial factors are potentially subject to ethnic, cultural, geographic, and economic factors. Moreover, health care and social systems vary by country. Additional local epidemiologic studies and international collaborative studies are needed to ensure effective integration of health care worldwide.

A series of epidemiologic studies of Japanese with rheumatoid arthritis (RA)^9^^,^^10^ and those on chronic hemodialysis^11^^–^^13^ examined the association between psychosocial factors and patient quality of life (QOL). The designs and major findings of these studies are summarized below.

## EPIDEMIOLOGIC STUDY OF PATIENTS WITH RHEUMATOID ARTHRITIS

RA is a chronic disease that causes inflammation of the joints and surrounding tissues. It is believed to be an autoimmune disorder; however, its etiology is not fully understood. Patients with RA have pain, stiffness, swelling, and destruction of the joints. Those with severe chronic disorders accompanied by pain, disability, and disfigurement have a higher risk of emotional disturbances^8^; therefore, it is not surprising that patients with RA are twice as likely as the general population to be depressed.^14^ Thus, the QOL of patients with RA is complicated with regard to the link between psychosocial and biological factors.

### Study design

We performed a cross-sectional epidemiologic study of the interrelationships between the psychosocial and physiological factors that determine the disease status of people with RA.^9^^,^^10^

In total, 213 patients (mean age, 60 years; range, 18–85 years) completed a series of health examinations and questionnaires. Disease severity, functional disability, counts of swollen and/or tender joints, duration of RA, frequency of arthritis surgery, and C-reactive protein (CRP) levels were assessed by rheumatologists. Self-report inventories completed by the patients were used to assess the perceived degree of pain and fatigue (visual analog scales), depression (Beck Depression Inventory-II^15^^,^^16^), anxiety (Hospital Anxiety and Depression Scale^17^), and social support (Social Support Questionnaire^18^^,^^19^). Mental and physical components of health-related QOL were evaluated using the Short Form-36 Health Survey.^20^^–^^23^

### Major findings

**Figure 1.**
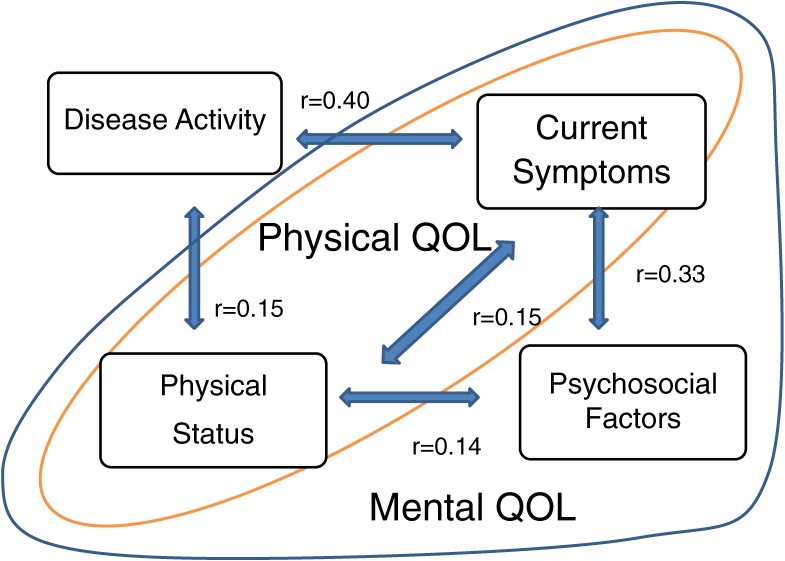
Interrelationships between psychosocial factors, disease activity, current symptoms, and physical status. The figure is based on the results of factor analysis of clinical and psychosocial data from 120 patients with rheumatoid arthritis. (Kojima M et al. J Psychosom Res. 2009;67(5):425–31. 2009, Elsevier Science Inc.)

Principal axis factor analysis revealed a 4-factor structure in which the components reflected psychosocial factors, disease activity, current symptoms, and physical functional status. Disease activity was independent of psychosocial factors and failed to reflect the perceived physical or mental QOL of patients with RA^10^ (Figure 1).

**Figure 2.**
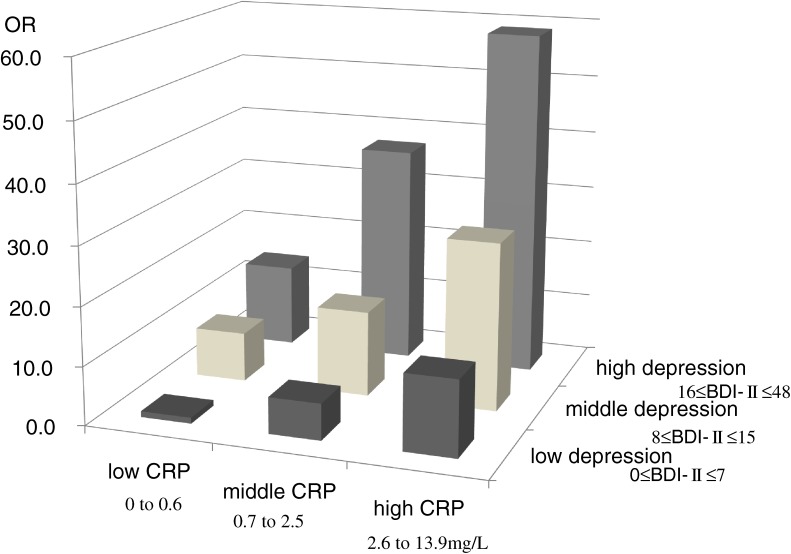
Impacts of depression and CRP on severe pain by tertiles of BDI-II score and CRP level. Using patients with a low BDI-II score and low CRP as the reference group, the odds ratios (ORs) for the presence of severe pain increased linearly with BDI-II score and CRP. (Kojima M et al. Arthritis Rheum. 2009;61:1018–24. 2009, American College of Rheumatology)

The associations among depression, pain, and inflammation were analyzed by multivariate analysis. Inflammation severity was evaluated by measuring the CRP level. Both depression score (standardized β = 0.35, *P* < 0.001) and CRP level (standardized β = 0.35, *P* < 0.001) were significantly associated with pain, even after adjusting for clinical covariates in the regression analysis. In logistic analysis, the combined effects on the risk of severe pain (pain score in the highest tertile) increased linearly with depression score and CRP level. Depression severity and inflammation were associated and appeared to have independent effects on perceived pain^9^ (Figure 2).

Clinicians should therefore evaluate psychosocial factors and subjective disease status to improve the QOL of patients with RA. A clinical approach that considers both the body and mind might be needed in order to achieve optimal pain control.

## EPIDEMIOLOGIC STUDY OF PATIENTS ON CHRONIC HEMODIALYSIS

Patients on chronic hemodialysis are at a high risk for emotional disturbances because of the burden due to illness, time constraints, diet restrictions, functional limitations, changes in self-perception, and fear of death. A positive association between depression and mortality has been reported in a population of such patients.^24^ Alexithymia is a personality construct that reflects a deficit in the cognitive processing of emotion.^25^ Alexithymic individuals tend to have difficulty identifying and describing their inner feelings, rarely fantasize, and have a utilitarian style of thinking. Alexithymia appears to be associated with various mental and physical health problems and to interfere with treatment compliance and treatment outcomes in clinical settings.^26^ A study of a large cohort of the Finnish general population reported that alexithymic men had a 2-fold risk for all-cause death (*P* < 0.001).^27^ However, it is not known if alexithymia is associated with other psychosocial factors and whether it influences long-term prognosis in patients on chronic hemodialysis.

### Study design

We hypothesized that depression and alexithymia would be independently associated with increased 5-year mortality among patients on chronic hemodialysis. We collected extensive psychosocial and clinical data at baseline to adjust for the influence of possible confounding factors.^11^^–^^13^

In total, 230 outpatients on hemodialysis (mean age, 56 years; range, 23–71 years) completed a battery of self-report measures, including the Beck Depression Inventory-II (BDI-II),^15^^,^^16^ 20-item Toronto Alexithymia Scale (TAS-20),^28^^,^^29^ Social Support Questionnaire,^18^^,^^19^ and Short Form-36 Health Survey.^20^^–^^23^ Laboratory data, including a 24-hour electrocardiogram, were also collected at baseline. Survival status was confirmed every 6 months for up to 5 years.

### Major findings

Baseline depression was significantly and independently associated with alexithymia (*P* = 0.004), and low satisfaction was associated with available social support (*P* = 0.01). Worsening of depressive symptoms after 6 months was predicted by alexithymia (adjusted odds ratio [OR], 2.6; 95% confidence interval [CI], 1.1–5.9) and social support (adjusted OR, 2.1; 95% CI, 1.0–4.4).^11^

Analysis of heart rate variability (HRV) and dynamics with the help of the 24-hour electrocardiogram (*n* = 119) revealed a clear association of depression with reduced HRV and loss of fractal HR dynamics.^12^

**Figure 3.**
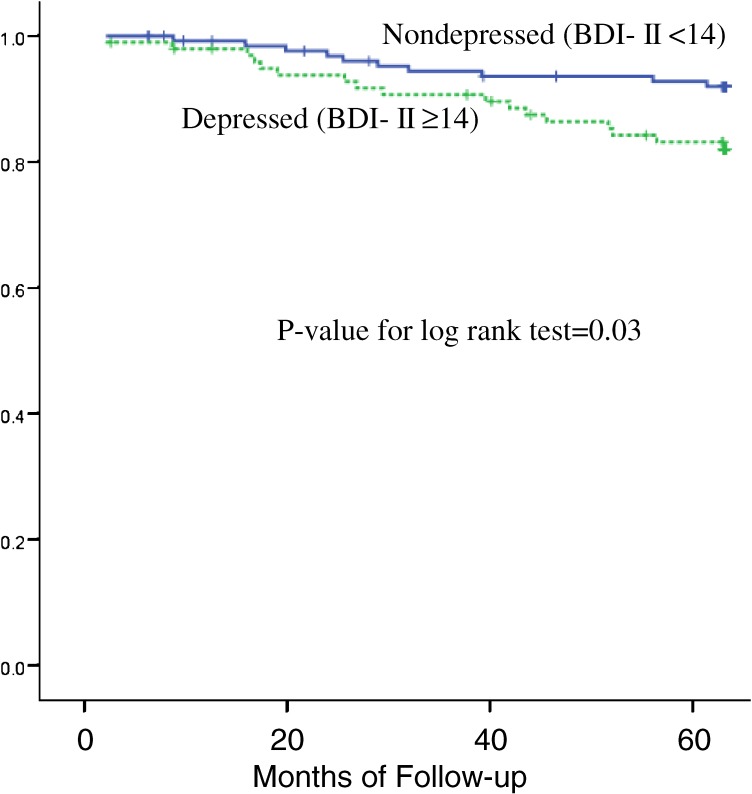
Kaplan-Meier survival curves by depression status. All-cause death-free survival by dichotomized level of BDI-II score in hemodialysis patients. (Kojima M et al. Psychother Psychosom. 2010;79:303–11. 2010, S. Karger AG, Basel)

**Figure 4.**
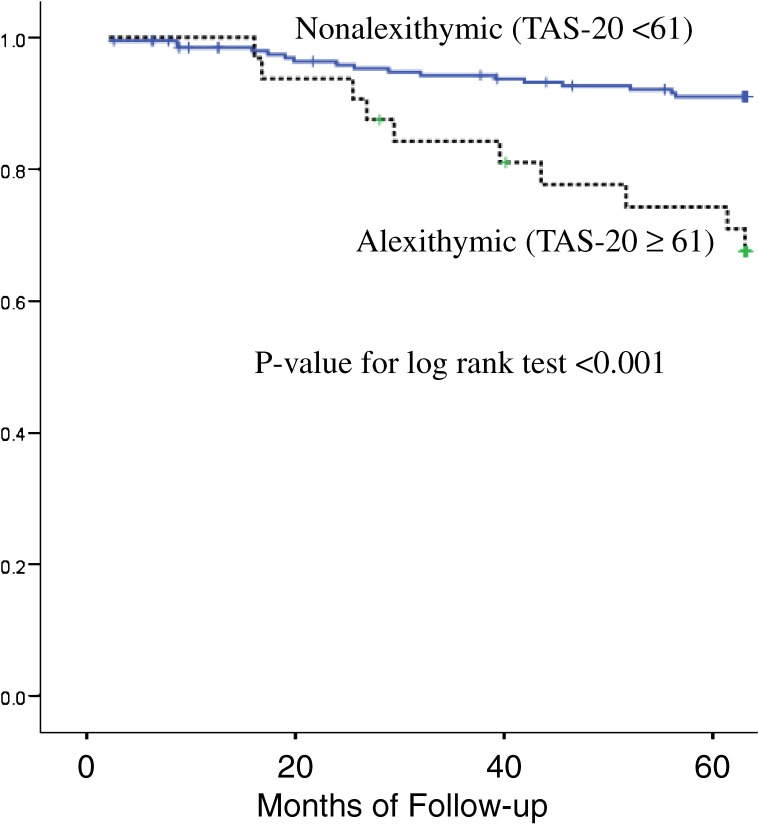
Kaplan-Meier survival curves by alexithymia status. All-cause death-free survival by dichotomized level of TAS-20 score in hemodialysis patients. (Kojima M et al. Psychother Psychosom. 2010;79:303–11. 2010, S. Karger AG, Basel)

Baseline depression and alexithymia were associated with an increased risk for all-cause 5-year mortality (Figures 3 and 4). However, only the association with alexithymia remained statistically significant after adjusting for baseline depression, health status (the SF-36 summary scores), marital status, and clinical covariates (multivariate adjusted hazard ratio, 3.62; 95% CI, 1.32–9.93; *P* = 0.01).^13^

Thus, depression, social support, and alexithymia were strongly associated and determined the QOL of patients on chronic hemodialysis (Table).

**Table.** Multivariate adjusted hazard ratios (HRs) for 5-year mortality associated with alexithymia and depression among 230 hemodialyzed patients

	Variables in model	Alexithymia			Depression			Change from previous step		
		TAS-20 ≥61			BDI-II ≥14								
		
		HR^a^	95% CI	*P* value	HR^b^	95% CI	*P* value	χ^2^	*df*^c^	*P* value
Model 1	Alexithymia, depression, age, and sex	3.54	1.55–8.11	0.003	1.75	0.77–3.99	0.18			
Model 2	Model 1 + PCS^d^ and MCS^e^ scores	3.64	1.48–8.96	0.005	2.13	0.86–5.23	0.10	7.86	2	0.02
Model 3	Model 2 + covariates^f^	3.62	1.32–9.93	0.012	1.70	0.64–4.48	0.29	15.90	6	0.01

^a^Hazard ratio shows increased mortality risk associated with presence of alexithymia (TAS-20 ≥61); ^b^Hazard ratio shows increased mortality risk associated with presence of depression (BDI-II ≥14); ^c^Degrees of freedom; ^d^Physical component summary score of SF-36; ^e^Mental component summary score of SF-36; ^f^Variables included in Model 3 as covariates were education ≥12 years, interdialytic weight gain, having comorbidity, hematoclit, calcium, and diastolic blood pressure. (Adapted from Kojima et al, “Depression, alexithymia and long-term mortality in chronic hemodialysis patients”, Psychotherapy and Psychosomatics 2010;79:303–11 2010 S. Karger AG, Basel.)

### Conclusion and future implications

Physiological, biological, and psychosocial factors are associated and determine our health independently and interactively. Epidemiology is a powerful tool for identifying effective points of intervention, after considering all possible confounders. Additional prospective studies are needed to identify variables that might be changed by intervention. We urgently need to develop effective psychosocial educational programs that improve the patient–doctor relationship and treatment outcomes and promote the health of the general population. Future studies are likely to clarify how we can improve our health by using a psychosocial approach.

## References

[1] Grad FP The Preamble of the Constitution of the World Health Organization. Bull World Health Organ. 2002;80(12):981–4 12571728 PMC2567708

[2] Williams RB The role of the brain in physical disease. Folklore, normal science, or paradigm shift? JAMA. 1990;263(14):1971–2 10.1001/jama.263.14.1971 2313874

[3] Engel GL The need for a new medical model: a challenge for biomedicine. Science. 1977;196(4286):129–36 10.1126/science.847460 847460

[4] Fassino S Psychosomatic approach is the new medicine tailored for patient personality with a focus on ethics, economy, and quality. Panminerva Med. 2010;52(3):249–64 21045782

[5] WHO. Global health risks: mortality and burden of disease attributable to selected major risks. Geneva: World Health organization; 2009.

[6] Prince M, Patel V, Saxena S, Maj M, Maselko J, Phillips MR, No health without mental health. Lancet. 2007;370(9590):859–77 10.1016/S0140-6736(07)61238-0 17804063

[7] Mathers CD, Loncar D Projections of global mortality and burden of disease from 2002 to 2030. PLoS Med. 2006;3(11):e442 10.1371/journal.pmed.0030442 17132052 PMC1664601

[8] Cohen S, Rodriquez MS Pathways linking affective disturbances and physical disorders. Health Psychol. 1995;14(5):374–80 10.1037/0278-6133.14.5.374 7498107

[9] Kojima M, Kojima T, Suzuki S, Oguchi T, Oba M, Tsuchiya H, Depression, inflammation, and pain in patients with rheumatoid arthritis. Arthritis Rheum. 2009;61(8):1018–24 10.1002/art.24647 19644894

[10] Kojima M, Kojima T, Ishiguro N, Oguchi T, Oba M, Tsuchiya H, Psychosocial factors, disease status, and quality of life in patients with rheumatoid arthritis. J Psychosom Res. 2009;67(5):425–31 10.1016/j.jpsychores.2009.01.001 19837205

[11] Kojima M, Hayano J, Tokudome S, Suzuki S, Ibuki K, Tomizawa H, Independent associations of alexithymia and social support with depression in hemodialysis patients. J Psychosom Res. 2007;63(4):349–56 10.1016/j.jpsychores.2007.04.002 17905041

[12] Kojima M, Hayano J, Suzuki S, Seno H, Kasuga H, Takahashi H, Depression, alexithymia and long-term mortality in chronic hemodialysis patients. Psychother Psychosom. 2010;79(5):303–11 10.1159/000319311 20664305

[13] Kojima M, Hayano J, Fukuta H, Sakata S, Mukai S, Ohte N, Loss of fractal heart rate dynamics in depressive hemodialysis patients. Psychosom Med. 2008;70(2):177–85 10.1097/PSY.0b013e31816477a1 18256338

[14] Ang DC, Choi H, Kroenke K, Wolfe F Comorbid depression is an independent risk factor for mortality in patients with rheumatoid arthritis. J Rheumatol. 2005;32(6):1013–9 15940760

[15] Beck AT, Steer RA. Manual for the Beck Depression Inventory-2. San Antonio, TX: Psychological Corporation; 1996.

[16] Kojima M, Furukawa TA, Takahashi H, Kawai M, Nagaya T, Tokudome S Cross-cultural validation of the Beck Depression Inventory-II in Japan. Psychiatry Res. 2002;110(3):291–9 10.1016/S0165-1781(02)00106-3 12127479

[17] Zigmond AS, Snaith RP The hospital anxiety and depression scale. Acta Psychiatr Scand. 1983;67(6):361–70 10.1111/j.1600-0447.1983.tb09716.x 6880820

[18] Furukawa TA, Harai H, Hirai T, Kitamura T, Takahashi K Social Support Questionnaire among psychiatric patients with various diagnoses and normal controls. Soc Psychiatry Psychiatr Epidemiol. 1999;34(4):216–22 10.1007/s001270050136 10365628

[19] Sarason BR, Levine HM, Basham RB, Sarason IG Assessing social support: the Social Support Questionnaire. J Pers Soc Psychol. 1983;44:127–39 10.1037/0022-3514.44.1.127

[20] Fukuhara S, Suzukamo Y. Manual of SF36v2 Japanese version. Kyoto: Institute for Health Outcomes & Process Evaluation Research; 2004.

[21] Fukuhara S, Ware JE Jr, Kosinski M, Wada S, Gandek B Psychometric and clinical tests of validity of the Japanese SF-36 Health Survey. J Clin Epidemiol. 1998;51(11):1045–53 10.1016/S0895-4356(98)00096-1 9817122

[22] Fukuhara S, Bito S, Green J, Hsiao A, Kurokawa K Translation, adaptation, and validation of the SF-36 Health Survey for use in Japan. J Clin Epidemiol. 1998;51(11):1037–44 10.1016/S0895-4356(98)00095-X 9817121

[23] Ware JE Jr, Sherbourne CD The MOS 36-item short-form health survey (SF-36). I. Conceptual framework and item selection. Med Care. 1992;30(6):473–83 10.1097/00005650-199206000-00002 1593914

[24] Hedayati SS, Bosworth HB, Briley LP, Sloane RJ, Pieper CF, Kimmel PL, Death or hospitalization of patients on chronic hemodialysis is associated with a physician-based diagnosis of depression. Kidney Int. 2008;74(7):930–6 10.1038/ki.2008.311 18580856

[25] Sifneos PE The prevalence of ‘alexithymic’ characteristics in psychosomatic patients. Psychother Psychosom. 1973;22(2):255–62 10.1159/000286529 4770536

[26] Taylor GJ, Bagby RM New trends in alexithymia research. Psychother Psychosom. 2004;73(2):68–77 10.1159/000075537 14767148

[27] Kauhanen J, Kaplan GA, Cohen RD, Julkunen J, Salonen JT Alexithymia and risk of death in middle-aged men. J Psychosom Res. 1996;41(6):541–9 10.1016/S0022-3999(96)00226-7 9032717

[28] Bagby RM, Parker JD, Taylor GJ The twenty-item Toronto Alexithymia Scale—I. Item selection and cross-validation of the factor structure. J Psychosom Res. 1994;38(1):23–32 10.1016/0022-3999(94)90005-1 8126686

[29] Bagby RM, Taylor GJ, Parker JD The Twenty-item Toronto Alexithymia Scale—II. Convergent, discriminant, and concurrent validity. J Psychosom Res. 1994;38(1):33–40 10.1016/0022-3999(94)90006-X 8126688

